# Genetic variability of the recombinant SARS-CoV-2 XEC: Is it a new evolutionary dead-end lineage?

**DOI:** 10.1016/j.nmni.2024.101520

**Published:** 2024-10-29

**Authors:** Francesco Branda, Massimo Ciccozzi, Fabio Scarpa

**Affiliations:** Unit of Medical Statistics and Molecular Epidemiology, Università Campus Bio-Medico di Roma, Rome, Italy; Department of Biomedical Sciences, University of Sassari, Sassari, Italy

**Keywords:** SARS-CoV-2 recombination, XEC variant, Genetic variability, Spike protein mutations, Evolutionary dead-end, Immune evasion

Dear Editor,

Recently, there has been an increase in SARS-CoV-2 cases, with many attributed to the new variant labeled XEC. This has raised concerns that, as is often the case with new variants, it could trigger new outbreaks and lead to critical situations. The XEC variant is currently a minor one, with its highest concentration in Germany, where the genomic prevalence is approximately 13% (https://www.epicov.org/epi3/frontend#227d24). In the UK, it makes up around 7%, while in the US, its prevalence is under 5% (https://www.epicov.org/epi3/frontend#227d24). Despite this, XEC seems to have a competitive growth edge, spreading more rapidly than other variants, indicating that it could become the predominant strain worldwide in the coming months. In this context, we have analyzed its genetic variability that define this new variant to better understand the actual risks posed by the spike protein mutations.

XEC originated from a recombination event between the two variants, KS.1.1 and KP.3.3. The two parental lineages are closely related, as both descended from JN.1, which was the globally dominant variant at the beginning of 2024 [1].

The recombination test conducted using the GARD (Genetic Algorithm for Recombination Detection) algorithm [2] confirms its formation as a recombinant between KS.1.1 and KP.3.3. The test indicated that the breakpoint occurred in the spike region, at a position between nucleotide site 800 and 900 (see Fig. 1). Since the two parental variants in question have a close evolutionary relationship, this region is quite conserved, with shared mutations present in both parental variants and the recombinant. For this reason, it was not possible to pinpoint the recombination point with greater detail. The first part of the XEC genome derives from KS.1.1, which thus acts as the acceptor in the recombination event, while KP.3.3 serves as the donor.

**Fig. 1:**
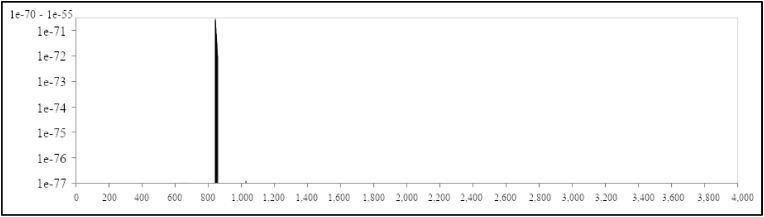
Model-averaged support for breakpoint placement estimated using the algorithm GARD. The X-axis indicates the nucleotide position in the spike protein at the recombination point, while the Y-axis represents the model-averaged support for the simulations performed using all available genomes in GISAID (https://gisaid.org). The figure has been edited using the software GIMP 2.10 (available at https://www.gimp.org).

One of the main concerns related to the potential dangers of XEC arises from two spike mutations inherited from its parental lineages, indeed, XEC carries the relatively rare T22N mutation (inherited from KS.1.1) and the Q493E mutation (from KP.3.3) in the spike protein.

However, while there is still limited knowledge about how the T22N mutation affects the virus's ability to replicate or spread due to its rarity, the Q493E mutation has been associated with increased binding affinity to the ACE2 receptor, potentially enhancing infectivity. This mutation may also influence the immune response, allowing for greater evasion by the immune system, particularly in individuals who are already infected or vaccinated.

The Q493E mutation might further impact the virus's ability to evade neutralizing antibodies. Its effects are often assessed alongside other mutations, as combinations within the receptor-binding domain (RBD) can produce synergistic effects, potentially increasing both the binding affinity to ACE2 and the capacity to evade the immune response [3].

The phylogenetic reconstruction based on the whole genome, conducted by means of nextstrain/ncov (https://github.com/nextstrain/ncov) using all currently available genomes from GISAID, indicates that individuals belonging to the recombinant XEC and its first descendant XEC.1 cluster within the clade containing both parental lineages (Fig. 2a). More specifically, they exhibit a genetic makeup closely related to the parental donor KP.3.3, clustering in clade 24C alongside it. Indeed, although as acceptor it has received a larger portion of the genome from KS.1.1, it shares the highest number of mutations with KP.3.3 across the entire genome [4], as also shown in the scatter plot in Fig. 2b.

**Fig. 2:**
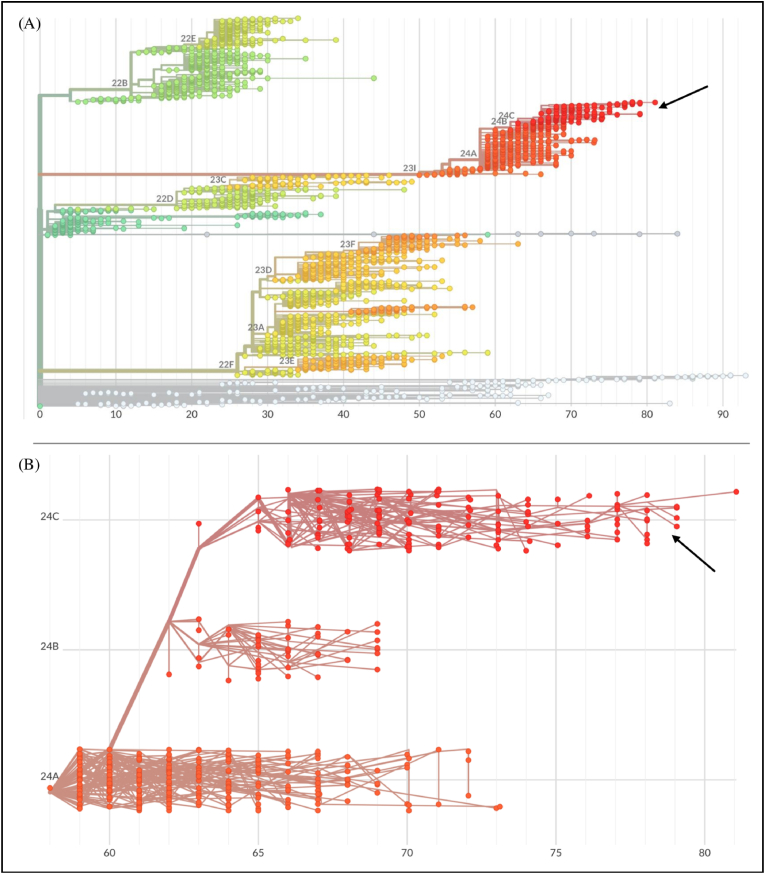
**(A)** Phylogenomic reconstruction by using all of the available genomes belonging to the most spread clades. The tree was created using nextstrain/ncov (https://github.com/nextstrain/ncov), with genomes filtered to ensure high quality and coverage. **(B)** Scatter plot including genomes from the main clade of all descendants of JN.1. Black arrows indicate the group to which the XEC and XEC.1 lineages belong. The figure was edited using GIMP 2.10 software (available at https://www.gimp.org).

This condition indicates that, from an evolutionary standpoint, the sites under selection are predominantly in the spike protein, as confirmed by the selection pressure test conducted with the FUBAR algorithm [5], suggesting that a significant portion of genetic variability is concentrated in this region. It is important to note that in terms of prevalence, barring any new mutations that may lead to further changes in the genetic makeup of the variant, XEC and its descendants appear to possess the characteristics to potentially replace the JN.1 variant, which is currently the most globally represented, reported by 139 countries (https://www.who.int/publications/m/item/covid-19-epidemiological-update-edition-171).

The phylogeny of the variants indeed shows that, like its parental lineages and other members of the clade, XEC is merely another evolutionary dead end, arising from a typical and very common evolutionary event. At present, it does not have the potential to expand significantly in terms of global population size. Even if it were to gain advantages in terms of newly acquired infectivity, this does not imply that it will become more symptomatic or lethal. In fact, since evolution tends to progress forward, the opposite should be expected. The emergence of recombinants and new variants is a normal biological process that will continue unabated.

The evolution of viruses, characterized by genomic changes, is a natural and ongoing process. These mutations occur as the virus adapts to its environment, including the host immune response and antiviral treatments. It is essential to recognize that an increase in genomic diversity does not inherently indicate a more severe or dangerous virus. While some mutations can confer advantages, such as enhanced transmissibility or immune evasion, many mutations have neutral or even detrimental effects on the virus's fitness. Consequently, the emergence of new variants should be viewed through a nuanced lens. In addition, T cells continue to provide significant protection against new variants, even in the face of some immune evasion by emerging lineages. This protection is maintained through immunological memory, strengthened by hybrid immunity from both vaccinations and previous infections. Consequently, an increase in a virus's fitness does not automatically imply a rise in contagion or risk.

Of course, this should not be interpreted as a reason to lower vigilance or discontinue constant monitoring. In fact, genome-based surveillance remains the only tool that allows for the immediate detection of evolutionary processes that could potentially lead to serious consequences.

## CRediT authorship contribution statement

**Francesco Branda:** Conceptualization, Investigation, Writing – original draft, Writing – review & editing. **Massimo Ciccozzi:** Conceptualization, Supervision, Validation, Writing – original draft, Writing – review & editing. **Fabio Scarpa:** Conceptualization, Data curation, Formal analysis, Investigation, Visualization, Writing – original draft, Writing – review & editing.

## Funding

None.

## Declaration of competing interest

The authors declare that they have no known competing financial interests or personal relationships that could have appeared to influence the work reported in this paper.
